# Osteoporosis Remission and New Bone Formation with Mesoporous Silica Nanoparticles

**DOI:** 10.1002/advs.202101107

**Published:** 2021-06-06

**Authors:** Patricia Mora‐Raimundo, Daniel Lozano, Manuel Benito, Francisca Mulero, Miguel Manzano, María Vallet‐Regí

**Affiliations:** ^1^ Chemistry in Pharmaceutical Sciences School of Pharmacy Universidad Complutense de Madrid Instituto de Investigación Sanitaria Hospital 12 de Octubre i + 12 Plaza de Ramón y Cajal s/n Madrid E‐28040 Spain; ^2^ Networking Research Center on Bioengineering Biomaterials and Nanomedicine (CIBER‐BBN) Madrid E‐28034 Spain; ^3^ Department of Biochemistry and Molecular Biology School of Pharmacy Universidad Complutense de Madrid Plaza de Ramón y Cajal s/n Madrid E‐28040 Spain; ^4^ Spanish Biomedical Research Centre in Diabetes and Associated Metabolic Disorders (CIBERDEM) Instituto de Salud Carlos III Madrid 28040 Spain; ^5^ Molecular Imaging Unit Spanish National Cancer Research Center (CNIO) Madrid E‐28029 Spain

**Keywords:** combination therapy, mesoporous silica nanoparticles, osteoporosis, osteostatin, small interfering RNAs

## Abstract

Nanotechnology changed the concept of treatment for a variety of diseases, producing a huge impact regarding drug and gene delivery. Among the different targeted diseases, osteoporosis has devastating clinical and economic consequences. Since current osteoporosis treatments present several side effects, new treatment approaches are needed. Recently, the application of small interfering RNA (siRNA) has become a promising alternative. Wnt/*β*‐catenin signaling pathway controls bone development and formation. This pathway is negatively regulated by sclerostin, which knock‐down through siRNA application would potentially promote bone formation. However, the major bottleneck for siRNA‐based treatments is the necessity of a delivery vector, bringing nanotechnology as a potential solution. Among the available nanocarriers, mesoporous silica nanoparticles (MSNs) have attracted great attention for intracellular delivery of siRNAs. The mesoporous structure of MSNs permits the delivery of siRNAs together with another biomolecule, achieving a combination therapy. Here, the effectiveness of a new potential osteoporosis treatment based on MSNs is evaluated. The proposed system is effective in delivering SOST siRNA and osteostatin through systemic injection to bone tissue. The nanoparticle administration produced an increase expression of osteogenic related genes improving the bone microarchitecture. The treated osteoporotic mice recovered values of a healthy situation approaching to osteoporosis remission.

## Graphical abstract

A mesoporous silica nanoparticles‐based system is designed for co‐delivering small interfering RNAs and an osteogenic peptide (osteostatin). The system is able to protect and deliver both biomolecules in the target tissue with promising results. It modifies gene expression and improves bone microarchitecture recovering healthy values. The application of nanoparticles can be considered a new potential alternative for osteoporosis remission.

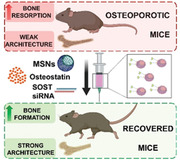

## 1 Introduction

Over the last 2 decades, engineered nanomaterials have been successfully tested and applied in medicine and pharmacology, especially for diagnostic or therapeutic purposes.^[^ ^1^, ^2^, ^3^ ^]^ In fact, nanoparticles have produced a huge impact as drug and gene delivery vehicles.^[^ ^2^ ^]^ Among them, mesoporous silica nanoparticles (MSNs) have attracted research interest in biomedicine due to their great properties for drug delivery such as, large surface area, high loading capacity due to their mesoporous structure, and biocompatibility.^[^ ^4^, ^5^, ^6^ ^]^ MSNs have been proposed for delivering different biomolecules against several diseases enhancing the efficacy of the treatment.^[^ ^7^ ^]^ They have been widely used to improve cytotoxic cancer treatments,^[^ ^8^, ^9^, ^10^ ^]^ antibacterial agents in infection scenarios^[^ ^11^, ^12^ ^]^ and antiosteoporotic therapies^[^ ^13^, ^14^ ^]^ among others.

Osteoporosis is considered one of the most common systemic skeletal disease.^[^ ^15^ ^]^ The prevalence of osteoporosis and its sequelae is rising substantially as a consequence of the increased life expectancy, becoming one of the largest global healthcare burdens.^[^ ^16^, ^17^ ^]^ The osteoporosis treatments mainly enclose orally administrated tablets or capsules (some bisphosphonates), subcutaneous injections (teriparatide) or intravenous perfusions (zoledronate), among others.^[^ ^15^, ^18^ ^]^ While these conventional drugs are effective, they present some limitations and side effects, such as osteonecrosis of the jaw or increased risk of breast cancer, which restrict their long‐term administration and adherence.^[^ ^19^ ^]^ For this reason, novel therapeutic targets for drug development and drug delivery systems for reducing off‐target effects have been investigated.^[^ ^13^, ^16^ ^]^

Wnt/*β*‐catenin signaling pathway plays an essential role in bone development and bone formation, regulating the proliferation, differentiation, and apoptosis of certain bone cells.^[^ ^20^, ^21^, ^22^ ^]^ Sclerostin and dickkopf‐1 proteins are the main inhibitors of this pathway reducing the activity of osteoblast, leading to a reduced bone formation.^[^ ^23^, ^24^, ^25^ ^]^ This fact leads to a potential therapeutic approach which could be blocking sclerostin action. In this sense, inhibition of sclerostin by monoclonal antibodies has shown effectiveness in restoring bone loss and increasing bone mass and strength.^[^ ^17^, ^26^, ^27^ ^]^ However, the anti‐sclerostin antibodies could produce an immune response, increasing blood clearance and preventing the potential new administrations.^[^ ^19^, ^28^ ^]^

RNA interference is a natural cellular process that regulates gene expression. It leads to the degradation of a specific messenger RNA (mRNA) knocking down the correspondent protein.^[^ ^29^ ^]^ Sclerostin is encoded by SOST gene, therefore silencing SOST through the delivery of small interfering RNA (siRNA) could reduce sclerostin expression,^[^ ^14^ ^]^ being an effective approach that may not be subjected to the antibody's limitations previously mentioned. However, siRNAs are well known by their short half‐life, being immediately degraded by RNases in the organism, and their poor penetration capability through cell membranes.^[^ ^30^ ^]^ These unresolved issues have inspired the use of nanocarriers to protect and deliver them.^[^ ^14^, ^31^, ^32^ ^]^ Among the different types of nanocarriers, MSNs have attracted research interest for nucleic acid delivery.^[^ ^33^, ^34^ ^]^ Although poly(ethylenimine) (PEI) is used as delivery vehicle itself,^[^ ^35^, ^36^, ^37^ ^]^ it could also be employed to decorate the nanoparticle surface for further plasmid complexation. In this sense, MSNs grafted with PEI could be used as siRNA nanocarriers, binding them on their surface, and encapsulating small molecules within the mesopores.^[^ ^14^, ^38^, ^39^, ^40^, ^41^ ^]^ This new platform permits the administration of two different therapeutics in the same nanocarrier. Combining agents with different mechanism of action (anabolic + antiresorpotive)^[^ ^42^, ^43^, ^44^, ^45^ ^]^ or two or more agents with the same effect but by different pathways (anabolic + anabolic)^[^ ^14^, ^46^ ^]^ could be an interesting strategy to confront the disease obtaining better results in osteoporosis remission.^[^ ^47^, ^48^, ^49^ ^]^ The combination therapy would be liked to exhibit synergistic or additive anti‐osteoporotic effects, but the reality proves that not every combination achieve it. We have previously reported that the combination of osteostatin, an osteogenic peptide derived from PTHrP,^[^ ^50^, ^51^, ^52^ ^]^ and SOST siRNA, both delivered by MSNs, produced a synergistic effect.^[^ ^14^ ^]^ The system was used as a local treatment, silencing SOST and increasing the expression of osteogenic markers in vivo through a bone marrow injection. Other local treatments have been studied as possible alternatives,^[^ ^53^ ^]^ but since the disease affects systematically to the organism, systemic approaches have been pursued. The chosen treatment should be intravenously administrated, preserving its stability and being accumulated in the target tissue, in this case, the bone. Bisphosphonates, such as alendronate (ALN), present a strong affinity toward hydroxyapatite (HA), the main component of mineralized bone matrix.^[^ ^54^ ^]^ Consequently, they have been used as bone targeting moiety to deliver therapeutics to the skeleton.^[^ ^55^ ^]^

**Figure 1:**
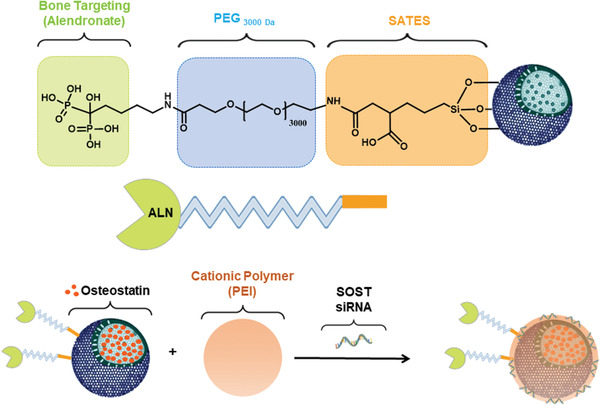
The nanoparticle‐based treatment approach. The surface of mesoporous silica nanoparticles was functionalized with an alendronate (ALN) modified poly(ethyleneglycol) (PEG). Nanoparticles were loaded with osteostatin, coated with the cationic polymer and bound to SOST siRNA leading to the final system. 3‐triethoxysilylpropylsuccinic anhydride (SATES).

Here, we have focused on the use of MSNs for osteoporotic combination treatment by delivering an osteogenic peptide and a siRNA plasmid. The system is based on MSNs modified with an ALN functionalized poly(ethyleneglycol) (PEG) ligand to increase stability and achieve bone targeting, and coated with PEI for the co‐delivery of SOST siRNA and osteostatin (**Figure** **1**). The final goal was the development of a potential systemic treatment for osteoporosis showing the relevance of nanotechnology for the improvement in bone diseases therapy. The development of this system could get us one step further for osteoporosis remission.

## 2 Results and Discussion

### 2.1 Nanoparticles Synthesis and Optimization

**Figure 2:**
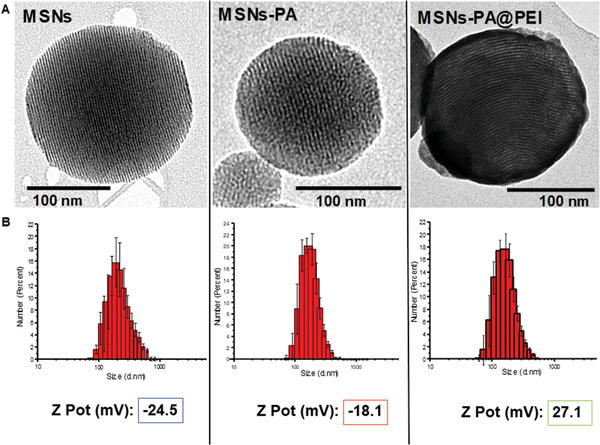
A) TEM micrographs, B) distribution and z potential of nanoparticles before and after grafting with the different polymers (PA and PEI).

The mesoporous silica nanoparticles were synthesized following a modification of the Stöber method,^[^ ^56^ ^]^ obtaining nanoparticles of ≈150 nm and mesopores of 2 nm. Poly(ethyleneglycol) was conjugated either to ALN to obtain the targeting ligand (PA), or to a residue of glycine to obtain the PEG‐NH_2_ (Scheme S1, Supporting Information). Both conjugates were used to decorate the nanoparticles surface, obtaining the two different functionalities pursued (see the Section 4 for details of MSNs synthesis, functionalization, and characterization). The PEGylated nanoparticles were further coated with PEI (MSNs‐PA@PEI). The correct functionalization of the particles was confirmed through transmission electron microscopy (TEM), dynamic light scattering (DLS), and z potential as it can be observed in **Figure** **2**.

Regarding TEM micrographs, the presence of a stained layer after phosphotungstic acid labeling only in the PEI coated nanoparticles confirms the coating with the polymer. PEI produced a polymeric matrix around the nanoparticle surface compared with the slight functionalization with PA (Figure 2A). The size distribution of the different nanoparticles was also explored through DLS analysis. Figure 2B showed that the monodispersity was unaffected by the successive reactions at their surface. Furthermore, it can be appreciated a change in the zeta potential after the different modifications of the surface (Figure 2B). Typical negative values of zeta potential for naked nanoparticles (−24 mV) were observed and after PA grafting, the zeta potential modestly increased up to −18 mV as a consequence of the condensation of silanol groups with PEG. However, after PEI coating, the zeta potential changed drastically to highly positive values rising to 27 mV. These data confirmed that the external surface of the particles was successfully functionalized (more details of the characterization can be found in the Supporting Information).

**Figure 3:**
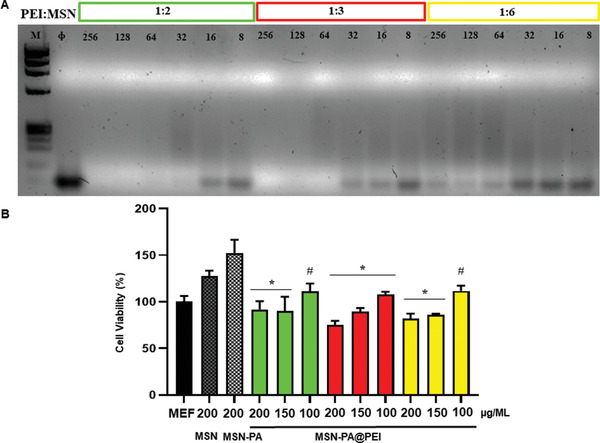
SiRNA binding to MSNs‐PA@PEI and cell viability of MC3T3‐E1 cells. A) Agarose gel electrophoresis of MSNs‐PA@PEI (PEI:MSNs‐PA ratio, 1:2 green, 1:3 red, 1:6 yellow) and complexed siRNA in different nanoparticle to nucleic acid (N/P) ratios (8–256). *M*: molecular weight marker. The *ϕ* lane contains only siRNA. B) MSNs‐PA@PEI cell viability. MC3T3‐E1 cell viability in contact with different concentrations of MSNs‐PA@PEI nanoparticles at 48 h of cell culture. Data are mean ± SEM of three independent experiments performed in triplicate (*n* = 3). Statistical significance was assessed by Kruskal–Wallis test and post hoc Dunn's test. Asterix indicates *p* < 0.01 versus MSNs and hashtag indicates *p* < 0.05 versus MSNs.

The amount of PEI grafted to the MSNs surface was optimized to achieve the maximum siRNA bound without comprising cell viability (**Figure** **3**). Different ratios of PEI:MSNs‐PA were compared (1:2 green, 1:3 red, 1:6 yellow).

To evaluate the binding capacity of the polymeric coating toward the nucleic acid, the highest amount of siRNA that could be bound to MSNs‐PA@PEI was determined by agarose gel electrophoresis (Figure 3A). Increasing amounts of MSNs‐PA@PEI were dispersed together with a constant amount of siRNA to obtain nanoparticle‐to‐nucleic acid ratios (N/P) of 8−256. The results observed in Figure 3A indicated that all siRNA was bound to the nanoparticles at an N/P ratio >32 for 1:2 ratio and >64 for 1:3 ratio. On the other hand, the 1:6 ratio was unable to properly bind siRNA. As a consequence, the 1:6 ratio was directly dismissed for further investigations.

The in vitro cell viability of MSNs‐PA@PEI was evaluated in a preosteoblastic cell line, MC3T3‐E1. As it can be appreciated in Figure 3B, MSNs‐PA@PEI of different ratios showed non‐toxic effects at concentrations as high as 200 µg mL^−1^ as it was the case of naked MSNs. Consequently, the MSNs‐PA@PEI 1:2 ratio resulted on the best loading capacity with non‐toxic effect being selected and further used for the next steps in our research.

The cellular uptake evaluation of the nanoparticles was performed in MC3T3‐E1 cells (Figure S5, Supporting Information). After coating the MSNs‐PA with PEI, the nanoparticle uptake significantly increased. This fact could be ascribed to the drastic change on the surface charge, from negative (MSNs‐PA) to positive (MSNs‐PA@PEI) promoting the folding of the nanoparticles by the cell membrane.

The next step to achieve the final system was the drug loading with the osteogenic peptide. The osteostatin release profile from the designed system was evaluated confirming that the cargo was released from the functionalized nanoparticles and PA and PEI coating were not acting as a physical barrier. Therefore, MSNs‐PA@PEI could be used to co‐transport and co‐deliver osteostatin and SOST siRNA simultaneously (Figure S6, Supporting Information).

The degradation produced in the final system over the days in different media, water, phosphate buffered saline (PBS) solution, Dulbecco's modified eagle's medium (DMEM) and ethanol, was evaluated in order to establish a conservation protocol of the designed nanocarrier from the moment of synthesis until administration. The election of these different media was regarding the composition of corporal medium (water, salts and proteins) to mimic the organism situation and another solvent no water based (ethanol). Figure S7, Supporting Information, shows how the nanoparticles were greatly degraded in water, PBS, or DMEM even after 24 h of contact, compared with the suspension in ethanol. In this case, the nanoparticles remained intact even after 15 days in contact with the solvent. Considering the results obtained, the nanoparticles conservation was performed in ethanol.

#### 2.1.1 Bone Targeting

**Figure 4:**
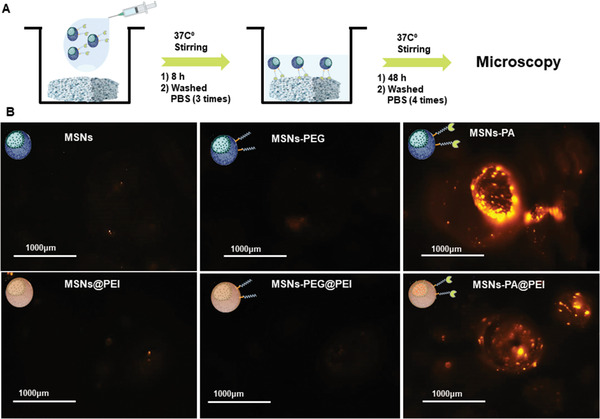
Hydroxyapatite retention behavior of MSNs‐PA@PEI. A) Hydroxyapatite retention assay. B) Representative microscopy images of HA tablets incubated for 8 h with Rhodamine‐B‐labeled (red fluorescence) MSNs, MSNs‐PEG, MSNs‐PA, MSNs@PEI, MSNs‐PEG@PEI, and MSNs‐PA@PEI nanoparticles.

The aim of functionalizing the surface with PA was to accomplish a bone targeting capacity to enhance the accumulation of nanoparticles around the bone tissue. A hydroxyapatite targeting assay was performed to evaluate the targeting capacity of the new system. The functionalized nanoparticles were exposed to a commercial HA tablet (ENGIPORE pre‐formed bone substitute) and then the retention capacity was evaluated (**Figure** **4**).

Different Rhodamine‐B‐labeled nanoparticles, MSNs, MSNs@PEI, MSNs‐PEG, MSNs‐PEG@PEI, MSNs‐PA, MSNs‐PA@PEI, were incubated with the HA tablet (Figure 4A). The ALN present in the PA residue of the functionalized nanoparticle surface exhibited high affinity toward HA, producing an increased nanoparticle accumulation in the HA tablet. In Figure 4B, it can be appreciated that only when the ALN of the PA polymer was on the MSNs surface, the nanoparticles were retained inside the HA matrix. This fact confirms the capacity of this new system to be accumulated by HA thanks to the presence of the bisphosphonate in the PEG conjugate as initially hypothesized.

#### 2.1.2 Colloidal Stability

**Figure 5:**
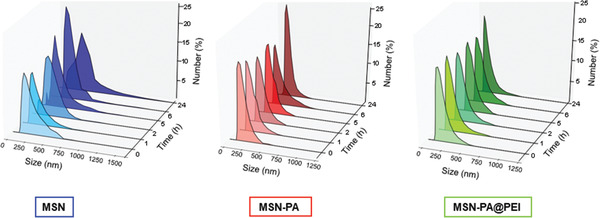
Colloidal stability of MSNs‐PA@PEI nanoparticles. Hydrodynamic diameter of MSNs (blue), MSNs‐PA (red), and MSNs‐PA@PEI (green) nanoparticles after 1, 2, 5, 6 and 24 h in PBS.

Decorating the nanoparticles with PEG aimed at the augmentation of the colloidal stability in suspension avoiding the nanoparticle aggregation. The stability of the PEGylated nanoparticles (MSNs‐PA) coated with PEI (MSNs‐PA@PEI) was compared with non‐PEGylated nanoparticles (MSNs) in PBS solution (**Figure** **5**).

The nanoparticle suspension stability was confirmed by evaluating the hydrodynamic diameter of the different nanoparticles after different periods of time in PBS (Figure 5). Non‐PEGylated nanoparticles were aggregated after 2 h in suspension, while the monodispersity of PEGylated nanoparticles remained unmodified for over 24 h, meaning that nanoparticle aggregation was not taking place. Then, the stability of the nanoparticle suspension in PBS was greatly improved in the PEGylated systems. It should be also remarked that the PEI coating after PEGylation did not affect to the stability of the nanoparticles. This enhancement was further evaluated by sedimentation of nanoparticles, confirming that MSNs were observed at the bottom of the cuvette after 1 h without stirring while MSNs‐PA or MSNs‐PA@PEI remained dispersed (Figure S8, Supporting Information).

#### 2.1.3 RNase Type I Stability

**Figure 6:**
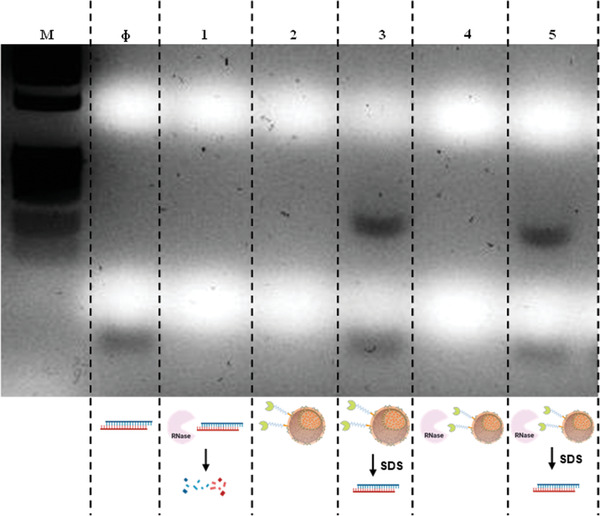
RNase type I stability assay. Agarose gel electrophoresis of MSNs‐PA@PEI‐siRNA exposed to RNase. *M*: molecular weight marker; *Φ*: free siRNA. Channel 1: free siRNA incubated for 2 h with RNase type I. Channel 2: MSNs‐PA@PEI bound with siRNA at 32 N/P. Channel 3: MSNs‐PA@PEI bound with siRNA and incubated with sodium dodecyl sulfate (SDS) for 15 min. Channel 4: MSNs‐PA@PEI bound with siRNA incubated with RNase type I for 2 h. Channel 5: MSNs‐PA@PEI bound with siRNA incubated with RNase type I for 2 h, then incubated with ethylenediaminetetraacetic acid (EDTA) for enzyme inactivation, and finally mixed with SDS for the display of siRNA.

One of the limitations of using free siRNA for systemic treatment is its rapid degradation by nucleases.^[^ ^31^ ^]^ To evaluate the loaded siRNA stability against RNase type I activity, a gel retardation assay was performed determining the integrity of siRNA after Rnase type I action (**Figure** **6**). Only uncomplexed and intact siRNA would appear at end of the gel. Therefore, either when siRNA is complexed to nanoparticles or it has been degraded by the RNase, the band would be no longer visible. Instead, only when siRNA resist the action of RNase type I, a band would appear at the end of the gel.

MSNs‐PA@PEI bound to siRNA were incubated with RNase type I and after inactivation of the enzyme, siRNA was released from the nanoparticles by the addition of sodium dodecyl sulfate (SDS), which displaced the siRNA from the polymer by competitive binding.^[^ ^57^ ^]^ In this case, a band appeared at the end of the gel, meaning that only when siRNA was loaded in the nanoparticles it resisted the action of RNase type I (Channel 5 Figure 6).

### 2.2 Nanoparticle Evaluation in Osteoporotic Mice

After the successful evaluation of the properties pursued for the nanoparticle system, the effect produced by the delivery of both molecules, SOST siRNA and osteostatin, was evaluated in a postmenopausal osteoporotic model. The most popular model for postmenopausal osteoporosis is conventionally obtained by the ovariectomy of female animals where the depletion of estrogen would produce significant bone loss after 14 days of surgery.^[^ ^58^ ^]^ Therefore, 12 weeks old ovariectomized C57/BL6 female mice, whose surgery was performed 6 weeks before, with a decreased femoral bone mineral density (BMD) compared to non‐ovariectomized (49.814 ± 0.28 mg cm^−3^ *versus* 46.67 ± 0.47 mg cm^−3^; *p* < 0.001) were here employed. They were exposed to the nanoparticles loaded both with SOST siRNA and osteostatin for 2 or 3 weeks. Nanoparticles were detectable inside mice for a limited period of time, which was determined to establish the frequency of the injections to be performed in the animals. (Figure S9, Supporting Information).

According to the obtained results, the injections of the complete system (OST‐MSNs‐PA@PEI‐siRNA) and each control (MSNs‐PA@PEI and free parathyroid hormone, PTH) were performed every 2 days for 2 and 3 weeks of treatment. Unloaded nanoparticles functionalized with PEG and ALN and covered with PEI (MSNs in the subsequent in vivo experiments) were evaluated as control to discard any potential effect of the employed nanocarrier (negative control). The current gold standard treatment for osteoporosis, PTH, was used to evaluate the better effectiveness of our nanosystem compared to an already approved treatment. The nanoparticles loaded with each drug alone were not evaluated since we confirmed the synergistic effect of SOST siRNA and osteostatin together in previous preliminary work and therefore we focus on the effect achieved by the delivery of both biomolecules together.^[^ ^14^ ^]^

Animals were euthanized after 2 or 3 weeks of treatment, respectively. Femur and tibiae were removed and analyzed by different techniques to evaluate the systemic effect.

**Figure 7:**
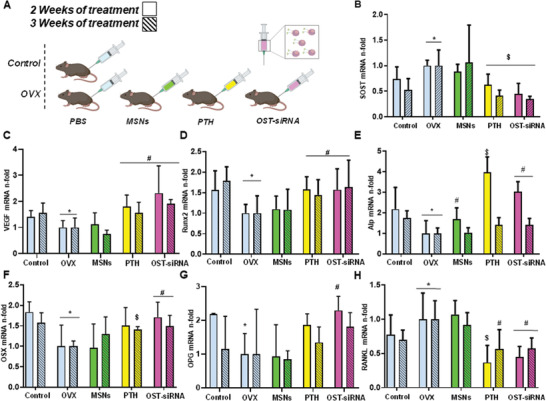
In vivo gene expression evaluation (measured by qRT‐PCR) in femur bone of healthy (Control) and ovariectomized (OVX) mice. In vivo assay scheme A) SOST, B) vascular endothelial growth factor (VEGF), C) Runx2, D) alkaline phosphatase (Alp), E) osterix (OSX), F) osteoprotegerin (OPG), G) receptor activator of nuclear factor κB ligand (RANKL), H) mRNA expression in presence of SOST‐siRNA and osteostatin loaded in MSNs‐PA@PEI nanoparticles (OST‐siRNA). A gold standard treatment control has been used (free PTH 100 µg Kg^−1^ every 2 days) (PTH), and free MSNs‐PA@PEI nanoparticles were used as negative control (MSNs). Data are mean ± SEM of five independent mice (*n* = 5). Statistical significance was assessed by Kruskal–Wallis test and post hoc Dunn's test. Asterisks indicate *p* < 0.05 versus Control; hashtag signs indicate *p* < 0.05 versus OVX, and dollar signs indicate *p* < 0.01 versus OVX.

The effect produced by the administrated system was evaluated through the expression of different osteoporotic genes such as SOST, Runx2, alkaline phosphatase (Alp), receptor activator of nuclear factor *κ*B ligand (RANKL), osteoprotegerin (OPG), osterix (OSX), and vascularization related genes such as vascular endothelial growth factor (VEGF). **Figure** **7** shows mRNAs measured by real‐time quantitative reverse transcription polymerase chain reaction (qRT‐PCR) after 2 (solid pattern) or 3 (line pattern) weeks of treatment.

SOST gene is involved in different developmental processes, particularly, inhibits osteoblastic activity and differentiation being responsible of bone formation modulation.^[^ ^22^, ^32^ ^]^ Therefore, its modification would affect bone status. In this study, the nanoparticles delivered SOST siRNA in order to reduce the expression of this gene and therefore increase bone formation. In Figure 7B, it can be appreciated that the expression of SOST in ovariectomized mice (OVX) is higher compared to non‐ovariectomized mice (Control). However, the injection of the nanoparticles with SOST siRNA and osteostatin (OST‐siRNA) notably decreased the expression of SOST after 2 or 3 weeks of treatment (≈ 50% and 60%, respectively). The systemic administration of the osteostatin and siRNA loaded nanoparticles decreased the expression of the target gene, verifying the knockdown activity of the injected system. Therefore, the synthesized nanoparticles were able to protect the siRNA and the peptide from the enzyme degradation, transporting the therapeutic agents to the target cells and producing the desired effect.

The expression of VEGF can be related to bone formation process, considering the well characterized relationship between angiogenesis and osteogenesis.^[^ ^59^ ^]^ In this sense, the expression of VEGF gene was increased after the administration of the nanoparticles (OST‐siRNA) (Figure 7C). This fact would be in agreement with the hypothesis stated before.

Different osteogenic markers are related with new bone formation. Since ovariectomy increases the bone resorption process, the expression of genes related with osteogenic features are expected to lessen. According to this, the osteogenic markers, Runx2 (Figure 7D), Alp (Figure 7E), OSX (Figure 7F), and OPG (Figure 7G) decreased their expression in OVX compared to control group. In this case, being SOST a gene involved in cell differentiation inhibition, its knockdown was expected to increase the expression of osteogenic markers.^[^ ^22^, ^32^, ^60^ ^]^ After the application of the system with SOST siRNA and osteostatin, the results showed that the expression of the genes notably increased, demonstrating the osteogenic feature of the designed system.

The RANK/RANKL/OPG regulatory axis plays a key role in the regulation of bone formation and resorption.^[^ ^61^, ^62^, ^63^ ^]^ The ratio between RANKL and OPG determines which action will take part. In this sense, RANKL will increase bone resorption, linking to its receptor in osteoclast, increasing their activity. On the other hand, OPG will link to RANKL avoiding its connection with RANK and the consequent osteoclast activation. Figure 7H shows how the expression of RANKL increased after ovariectomy, but after the nanoparticles administration decreased, being in total agreement with the augmentation of OPG expression previously mentioned.

The effect achieved by the administration of our co‐loaded nanoparticles reached similar values of gene silencing and stimulation to the injection of free PTH (conventional osteoporosis treatment). The effect was even slightly higher by the application of the complete system in some particular cases such as OPG, VEGF, and OSX expression. Therefore, the system proposed here yielded better results than the gold standard therapy, PTH, being considered as a potential alternative for osteoporosis treatment.

**Figure 8:**
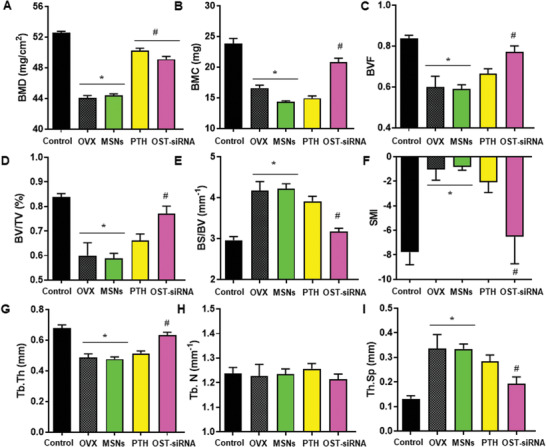
Representative parameters of trabecular morphology measured by µCT to evaluate the effect after 3 weeks of treatment. A) Bone mineral density (BMD); B) bone mineral content (BMC); C) bone volume fraction (BVF); D) trabecular bone volume per total volume (BV/TV); E) specific bone surface (BS/BV); F) structure model index (SMI); G) mean trabecular thickness (Tb.Th); H) mean trabecular number (Tb.N); I) mean trabecular separation (Tb.Sp). Data are mean ± SEM of five independent mice (*n* = 5). Statistical significance was assessed by Kruskal–Wallis test and post hoc Dunn's test. Asterisks indicate *p* < 0.05 versus Control and hashtag signs indicate *p* < 0.05 versus OVX.

Once our system was proven to modify gene expression, the next step consisted in evaluating its effect in bone microarchitecture. The ability of a bone to resist fracture depends not only on the bone mass, but also on its spatial distribution (macro‐ and microarchitecture), and the intrinsic properties of bone tissue. Thus, knowledge of bone microarchitecture is essential for understanding the pathophysiology of osteoporosis, determining the quality of bone, predicting fractures, and evaluating the efficacy of the treatment.^[^ ^64^ ^]^ The response of microarchitectural parameters to treatment should assess the real efficacy of anti‐osteoporotic treatments, which are primarily expected to stop the progression of disease and prevent fractures. For this reason, the architecture of the treated and non‐treated bones was evaluated by micro‐computed tomography (µCT) (**Figure** **8**). Since the most pronounced gene fluctuation was produced after 2 weeks, the architecture of the bone was evaluated after 3 weeks of treatment. In this sense, we would be able to detect the consequences of the gene expression modifications 1 week after in the bone microarchitecture.

The BMD is one of the most common used values to determine the quality of bone. It relates the amount of mineral (mainly HA) per volume of bone tissue. In osteoporotic‐like scenarios, BMD tent to decrease in comparison with healthy situation (control). In this case, BMD of healthy mice was ≈53 mg cm^−3^ (Control). Instead, ovariectomized mice presented lower value of BMD, ≈44 mg cm^−3^. Figure 8A shows that after 3 weeks of treatment the values of BMD increased ≈0% with PTH and ≈60% with our system (OST‐siRNA), approaching to a healthy situation.

The bone mineral content (BMC) was also evaluated, which expresses the amount of mineral in a specific area of the bone tissue.^[^ ^64^ ^]^ In this case, BMC also decreased after ovariectomy as expected. After the loaded nanoparticles administration (OST‐siRNA), BMC was recovered ≈65%. In this case, our system presented better results than PTH treatment (Figure 8B).

The basic morphometric indices include the measurement of bone volume (BV) and the tissue volume (TV). The ratio of these two measures is the bone volume fraction (BV/TV or BVF). Figure 8C,D shows that ovariectomized mice presented lower values of bone volume fraction compared to healthy mice. After 3 weeks of several injections of free PTH or OST‐MSNs‐PA@PEI‐siRNA (OST‐siRNA), both values increased recovering over 70% of the bone volume fraction after the nanoparticles administration and the 25% in the case of PTH treatment.

Another basic measure is the bone surface (BS), which is necessary to obtain the specific bone surface or bone surface fraction (BS/BV). Figure 8E shows that BS/BV increased considerably in the ovariectomized group, reducing its value after our system treatment (≈85%), reaching similar values to healthy mice, and after PTH application (≈20%).

An index called structure model index (SMI) was developed to estimate the trabecular bone structure. When SMI increases, indicates a worse bone structure, related with an osteoporotic scenario. In this case, after ovariectomy surgery in mice, the SMI increased from −8 to −1, and after treating mice with our system or with PTH, this value decreased again to values of −6 and −2 respectively (Figure 8F). The application of our system achieved better results than the obtained by the administration of PTH showing the potential of this new approach.

As noted earlier, µCT permits the evaluation of trabecular morphology by 3D measurements. Among them, the mean trabecular thickness (Tb.Th) tends to decrease after ovariectomy, leading to weaker bones. Figure 8G shows that Tb.Th decreased in ovariectomized mice, as expected, being recovered ≈75% by our nanosystem injections and ≈15% with PTH treatment. Related with this value, other measures such as the mean trabecular separation (Tb.Sp), or the mean trabecular number (Tb.N) were also measured. The Tb.Sp is the mean distance between trabeculae, therefore, is inversely proportional to Tb.Th, so in this case the value is expected to increase in ovariectomized mice. In Figure 8I it could be appreciated how Tb.Sp triplicates in the case of OVX compared with control group. After treatment either with our nanoparticles or with PTH the value decreased ≈70% or ≈25% respectively, showing the greater effectivity of the nanoparticles regarding the trabeculae structure. On the other hand, Tb.N, which is the average number of trabeculae per unit of length, did not present variations after either ovariectomy surgery or different treatments (Figure 8H). The number of trabeculae did not change even after ovariectomy, therefore osteoporosis led to a situation of thinner trabeculae but maintaining the same number. The application of the different treatments recovered the thickness lost during the disease progression.

Additionally, cortical region indices were evaluated. The changes observed were not as noticeable as those obtained in the trabecular region, but this could be related to the osteoporotic model used. Ovariectomized mice present the greater reduction of bone in the trabecular regions instead of the cortical section after 14 days from the surgery.^[^ ^58^ ^]^ Despite this fact, the cortical BMD was ≈60 mg cm^−3^ in healthy mice and decreased to ≈48 mg cm^−3^ after ovariectomy. After treatment either with our system or with PTH, this value increased to ≈58 or 50 mg cm^−3^, respectively. The cortical thickness (Ct. Th) was also measured, decreasing after ovariectomy from 0.87 to 0.75 mm. After treatment, this value was recovered ≈60% in the case of our system injections and ≈11% after PTH application. The application of the co‐loaded nanoparticles produced a greater effect compared with the gold standard osteoporosis treatment also in the cortical section. This trend has been repeatedly observed through several experiments, highlighting the benefits of the dual treatment with our MSNs nanocarriers.

**Figure 9:**
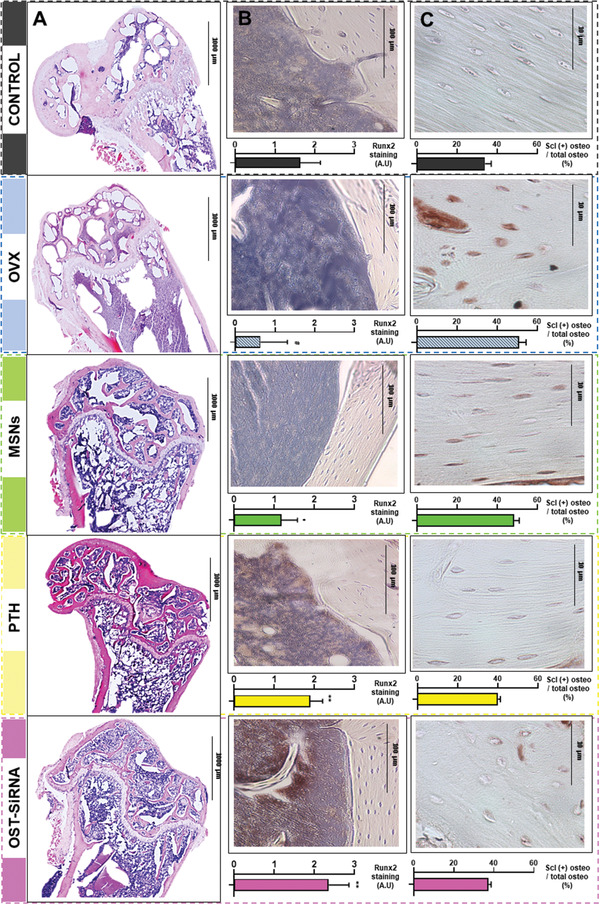
Histological analysis and immunostaining for Runx2 and sclerostin in the femur of mice. Representative images of the different femur histological sections after hematoxylin/eosin and Masson–Goldner Trichrome staining. A) Representative Runx2 immunostaining in mice femurs. Images show abundant positivity (brown stain) for the transcription factor in cells after PTH or OST‐siRNA treatments. B) Total and sclerostin‐positive osteocytes in the cortical femur. C) Data are mean ± SEM of five independent mice (*n* = 5). Statistical significance was assessed by Kruskal–Wallis test and post hoc Dunn's test. Hashtag indicates *p* < 0.001 versus Control, asterisk indicates *p* < 0.05 versus OVX, and double asterisk indicates *p* < 0.001 versus OVX.

To further evaluate the response of the bone to the different treatments, we performed histological and immunohistochemical analysis on the bone tissue after 3 weeks of treatment (**Figure** **9**).

As appreciated in Figure 9A, a reduction of bone and trabeculae was evident in the OVX group. On the other hand, after the administration of either empty MSNs, PTH, or our complete system (OST‐siRNA), an increment of bone formation was appreciated. This fact means that the presence of silica in the bone tissue possibly affects bone formation somehow, although it was not appreciated in the previous experiments of gene expression modification or in bone microarchitecture. In the case of PTH and OST‐siRNA the bone formation was greatly appreciated in the epiphysis of the bone, as shown in Figure 9A by the appearance of abundant osteoid and new trabeculae recovering a huge amount of the bone lost after ovariectomy.

The bone formation induction was further assessed by immunostaining transcription factors such as Runx2. The number of Runx2‐positive cells (brown stain) was reduced after ovariectomy as expected (Figure 9B). On the other hand, after the administration of either MSNs, PTH, or the combination of osteostatin and SOST siRNA, an increase of Runx2‐positive cells was achieved. Moreover, the greatest increase was achieved by the administration of our complete system in agreement with the previously mentioned results. Furthermore, the total and sclerostin‐positive osteocytes were measured by immunostaining in the femur cortical section (Figure 9C). The number of sclerostin‐positive osteocytes per number of osteocytes increased after ovariectomy. Instead, after 3 weeks of treatment with either PTH or our complete system, the reduction of sclerostin‐positive osteocytes was reduced to almost healthy values.

Then, we have been able to show that the administration of MSNs loaded with osteostatin and SOST siRNA exert a clear bone regeneration action as evaluated by the gene expression modification and histological, immunohistochemical and µCT analysis in OVX mice. The effect produced by the administration of our system was even higher than the effect generated by the administration of the conventional treatment, PTH. Therefore, the designed system represents a potential alternative to the current gold standard treatment for the osteoporosis.

## 3 Conclusions

Here, we have proposed a modified mesoporous silica nanoparticle‐based system able to transport and deliver SOST siRNA and osteostatin by subcutaneous injection. MSNs have been modified with PEG and ALN to confer the nanoparticles good colloidal stability and bone targeting capacity to deliver the biomolecules more effectively at the targeted tissue.

The targeting ability and the colloidal stability of the optimized system were validated. The system was able to protect siRNA from RNases, maintaining the monodispersity and suspension stability, and it was accumulated around synthetic HA.

The nanoparticles were loaded with osteostatin and SOST siRNA and injected subcutaneously in ovariectomized mice. The gene expression was modified after the administration of the nanoparticles, being SOST effectively knocked‐down, and important osteogenic markers and a vascularization related gene increased, being the first sign of new bone formation. This in vivo gene expression analysis demonstrated the efficiency of our system toward a potential osteoporosis treatment. The microarchitecture of the bones was also measured by µCT, histological, and immunohistochemical analysis. The results demonstrated that after 3 weeks of systemic treatment the microarchitecture of the bone was improved due to our loaded nanocarrier. Moreover, the application of the designed nanoparticles was compared with the effect achieved by one of the conventional treatments, PTH. The co‐delivery of both therapeutic agents from the nanoparticles not only achieved a greater stimulation of osteogenic markers compared to the PTH administration, but also the gene expression modification led to a better bone architecture compared with the hormone treatment. The treated ovariectomized mice almost recovered values of healthy mice, meaning that the here proposed system could be considered as a promising alternative for osteoporosis systemic treatment. It presented a new potential approach for osteoporosis remission yielding better results than the current gold standard anabolic treatment, PTH.

## 4 Experimental Section

### Synthesis of Mesoporous Silica Nanoparticles

Mesoporous silica nanoparticles were synthesized following a modification of the Stöber method.^[^ ^56^ ^]^ Cetyltrimethylammonium bromide (1 g, 2.74 mmol), the structure directing agent, was dissolved in H_2_O (480 mL) and NaOH (3.5 mL, 2 m) in a 1 L round‐bottom flask under moderate magnetic stirring. The mixture was heated for 20 min at 80 °C and then, tetraethyl orthosilicate (TEOS (4.5 mL, 20.15 mmol)), the silica precursor, was added drop‐wise at 0.33 mL min^−1^ rate with a pump. After 30 min of TEOS addition, 3‐trihydroxysilylpropyl methylphosphonate (0.5 mL, 1.31 mmol) was added for the phosphonate modification and heated for a further 1.5 h at 80 °C under magnetic stirring. Then, the solution was centrifuged and washed once with water and twice with ethanol. The product was dried at room temperature under vacuum obtaining a white powder.

The surfactant was removed by ionic exchange using a solution of ammonium nitrate (NH_4_NO_3_) (350 mL 10 mg mL^−1^) in ethanol (95%) at 80 °C overnight under magnetic stirring. The process was repeated for 2 h. Then, the product was centrifuged, washed 3 times with ethanol and dried under vacuum.^[^ ^65^ ^]^

Fluorescein isothiocyanate (FITC)‐labeled nanoparticles and Rhodamine‐B isothiocyanate‐labeled nanoparticles were synthesized by reacting FITC (1 mg) or Rhodamine‐B isothiocyanate (1 mg) with of 3‐aminopropyltriethoxysilane (APTES) (2.2 mL 0.009 mmol) in ethanol (40 µL) for 2 h at room temperature.^[^ ^66^ ^]^ Then, this solution was mixed with TEOS (4.5 mL), and the whole mixture was added to the surfactant solution as previously described. The rest of the procedure (silica precursor, functionalization and surfactant removal) was carried out as described above. For the synthesis of cyanine‐7(Cy7)‐labeled nanoparticles, Cy7 (5 mg) with APTES (4.4 µL) were dissolved in of dimethyl sulfoxide (DMSO) (140 µL) and stirred overnight at room temperature. Then, this solution was mixed with TEOS (4.5 mL), and the whole mixture was added to the surfactant solution as previously described. The rest of the procedure was carried out as described above.

The as‐produced particles were characterized in terms of X‐ray diffraction, Fourier transformed infrared spectroscopy, thermogravimetric analysis, N_2_ adsorption analysis, TEM, DLS, and zeta potential (Supporting Information).

### Synthesis and Characterization of PEG‐Alendronate Conjugate

In a N_2_‐purged flask, a commercial *O*‐[2‐(Boc‐amino) ethyl]‐*O*′‐[3‐(*N*‐succinimidyloxy)‐3‐oxopropyl] poly(ethylene glycol) (3500 Da) (153 mg) and sodium alendronate (30 mg) were dissolved in 100 µL of a mixture of DMSO/H_2_O 60:40 at room temperature.^[^ ^9^ ^]^ Triethylamine (30 µL) was added and the temperature was increased to 50 °C. The reaction was magnetically stirred overnight under inert atmosphere. The product (Boc‐NH‐PA) was precipitated in cold ether and purified by flash molecular exclusion chromatography (G‐25/H_2_O). The obtained product was characterized by ^1^H‐NMR (Supporting Information).

Boc‐NH‐PA was solved in a solution of dichloromethane/trifluoroacetic acid (DCM/TFA) 1% to remove the protecting Boc from the amino group. The mixture was stirred at room temperature for 1 h. The product was obtained by precipitation in cold ether. The obtained product (PA) was characterized by ^1^H‐NMR (Supporting Information).

### Synthesis and Characterization of Amine Functionalized PEG

Glycine amino group was functionalized with fluorenylmethyloxycarbonyl (Fmoc) protecting group, to avoid cross reactions between different molecules of amino acid. PEG‐methyl ether (2000 Da) (80 mg) and glycine‐Fmoc (24 mg) were solved in a mixture of dried DMSO (0.1 mL)/CH_2_Cl_2_ (2 mL). *N*,*N*′‐dicyclohexylcarbodiimide (DCC) (20 mg) and 4‐dimethylaminopyridine (DMAP) (2 mg, 0.015 mmol) were solved in dried CH_2_Cl_2_ (0.5 mL) and added dropwise the PEG solution. The reaction was performed in presence of DCC for the acid activation of the glycine, and with DMAP as catalyst. The reaction was magnetically stirred overnight. Finally, the alcohol group of PEG reacted with the activated acid of the glycine, obtaining the desired amino functionalized PEG. The product was precipitated in cold ether, solved in *N*,*N*‐dimethylformamide (DMF)/piperidine 20% and stirred for 2 h for the Fmoc amino group deprotection from the glycine. The obtained product was characterized by ^1^H‐NMR (Supporting Information)

### Poly(ethylene glycol) and Alendronate Modified Poly(ethylene glycol) Grafting

PA (1.5 mg) and PEG (1.5 mg) were added to a glass vial. The vial was purged with N_2_ and 1 mL of dry toluene was added. Then, dry toluene (0.5 mL) with 1 µL of 3‐triethoxysilylpropylsuccinic anhydride (SATES) was added dropwise to PEG mixture under N_2_ atmosphere and with magnetic stirring. A catalytic amount of DMAP was solved in 1 mL of toluene and finally added to the whole solution. The reaction was stirred overnight (Solution 1, sililated polymers solution).

Then, 1 mL of Solution 1 was added dropwise to toluene (5 mL) containing previously dispersed MSNs (50 mg) under vigorous stirring. The reaction medium was heated (80 °C) under reflux. After 1 h, the remaining Solution 1 was added. The reaction was left under vigorous stirring for 24 h. Then, the functionalized MSNs were collected by centrifugation and washed with toluene (twice) and ethanol (twice). Afterward, the nanoparticles (MSNs‐PA) were dried under vacuum for 16 h.

The same procedure was used to functionalize the nanoparticles only with PEG to obtain PEGylated nanoparticles (MSNs‐PEG) for experiment controls. PEG (3 mg) were solved in dry toluene under N_2_ atmosphere. Then, dry toluene (0.5 mL) with 1 µL SATES were added dropwise to PEG mixture with magnetic stirring. A catalytic amount of DMAP was solved in toluene (1 mL) and finally added to the whole solution. The reaction was stirred overnight. The same protocol described above was followed for the sililated polymer condensation onto the nanoparticle surface.

### Poly(ethylenimine) Grafting

Different nanoparticles, MSNs‐PA or MSNs‐PEG (5 mg) nanoparticles were dispersed in a solution of poly(ethylenimine) (PEI) (5 kDa) (2.5 mg) in absolute ethanol (1 mL) to perform the PEI coating. After sonicating for 20 s and stirring for 30 min,^[^ ^39^ ^]^ the PEI coated nanoparticles (MSNs‐PA@PEI, MSNs‐PEG@PEI) were consequently washed with PBS and ethanol. The amount of MSNs was increased to achieve the different PEI:MSNs ratios needed. It was performed 1:1 and 1:2 mass ratio for MSNs@PEI, and 1:2, 1:3, 1:6 for MSNs‐PA@PEI.

### SiRNA Binding Capability

The transfection conditions and the release efficiency of the siRNA from nanoparticles were optimized using a siRNA‐analog called siGLO Green Transfection Indicator (Abs/Em max 494/520 nm). The siRNA binding capability of MSNs‐PA@PEI was determined by agarose gel retardation assay. The three different ratios of MSNs‐PA@PEI were compared (1:2, 1:3 and 1:6). Different amounts of MSNs‐PA@PEI ranging from 0.8 to 25.6 µg were mixed with 0.1 µg of siGLO in aqueous solution to obtain nanoparticle to nucleic acid ratios (N/P) of 8−*256*. N/P is a mass ratio in which N and P, respectively, correspond to the positive (nitrogen (MSNs‐PA@PEI)) and negative (phosphonate (siGLO)) charges. Free siGLO was used as control. A total of 20 µL of MSNs‐PA@PEI and siGLO complex solution were mixed with 5 µL of loading buffer and electrophoresed in a 2% agarose gel containing 0.5 µg mL^−1^ of GelRed (Nucleic Acid Gel Stain) at 80 V for 40 min in Tris/Borate/ ethylenediaminetetraacetic acid (EDTA) running buffer. Nucleic acid bands were detected by ultraviolet (UV) light (254 nm). It is important to mention that only uncomplexed siGLO was able to migrate to the positive electrode and, therefore, be observed on the gel. Then, when the spot of free siGLO was no longer visible means that the amount of nanoparticles added had complexed with all the siGLO present (total binding particle/nucleic acid). The lowest N/P ratio that complexed all the siGLO is called threshold. For MSNs‐PA@PEI 1:2 the threshold was 34, for 1:3 ratio was 64, and 1:6 ratio was not able to bind properly the siGLO therefore the nucleic acid band did not disappear even in 256 N/P ratio.

### Cell Viability

Cell culture tests were performed using MC3T3‐E1 cells. Cells were plated at a density of 10^4^ cells per square centimeter in 1 mL of DMEM, containing 10% of heat‐inactivated fetal bovine serum and 1% penicillin−streptomycin at 37 °C in a humidified atmosphere of 5% CO_2_, and incubated for the specific time of each experiment. The tested nanoparticles were placed into each well of 12 or 24‐well plates after cell seeding. Some wells without nanoparticles were seeded as controls.

MC3T3‐E1 viability was determined by addition of Alamar Blue solution at 10% (v/v) to the cell culture. After 2 h of contact with different concentrations of MSNs (*n* = 3), the cells were grown for 48 h in 24 well plates. Afterward, Alamar Blue solution was added following the manufacturer's instructions. Fluorescence intensity was measured using excitation emission wavelengths of 570 and 600 nm, respectively, in a Unicam UV‐500 UV−vis spectrophotometer.

### Cell MSNs‐PA@PEI Uptake

MC3T3‐E1 cells were cultured in each well of a 12‐well plate and incubated at different times in the absence or presence of the tested FITC‐labeled nanoparticles at a concentration of 50 µg mL^−1^ (MSNs, MSNs‐PA, MSNs‐PA@PEI, and MSNs‐PA@PEI‐siGLO). After 2 h, cells were washed twice with PBS and incubated at 37 °C with trypsin−EDTA solution for cell detachment. The reaction was stopped with culture medium after 5 min, and cells were centrifuged at 1000 rpm for 10 min and suspended in fresh medium. Then, the fluorescence present in the surface of the cells was quenched with Trypan blue (0.4%) to confirm the presence of intracellular and, therefore, internalized fluorescent signal.

Flow cytometry measurements were performed at an excitation wavelength of 488 nm, and green fluorescence was measured at 530 nm (FL1). The trigger was set for the green fluorescence channel (FL1). The conditions for the data acquisition and analysis were established using negative and positive controls with the CellQuest Program of Becton−Dickinson, and these conditions were maintained during all the experiments. Each experiment was carried out three times and single representative experiments were displayed. For statistical significance, at least 10^4^ cells were analyzed in each sample in a FACScan machine (Becton, Dickinson and Company) and the mean of the fluorescence emitted by these single cells was used.

### Targeting Assay

To evaluate the targeting capacity of the nanoparticles, 500 µL of a solution of 1mg mL^−1^ of the different Rhodamine‐B‐labeled nanoparticles (MSNs, MSNs‐PA, MSNs@PEI MSNs‐PA@PEI) in PBS 1X (pH = 7.4) were incubated with a commercial HA (50 mg) tablet (ENGIPORE pre‐formed bone substitute) at 37 °C and 100 rpm during 8 h. The tablet was washed 3 times in PBS 1X (1.5 mL) and incubated for further 48 h at 37 °C and 100 rpm with fresh PBS 1X to wash the nanoparticles retained inside the tablet due to its porosity nature. Then, the piece was washed 4 times with fresh PBS 1X (1 mL). The tablets were analyzed by fluorescence microscopy to determine the presence of Rhodamine B‐labeled nanoparticles.

### Colloidal Stability Assay

To determine the stability of the different nanoparticles, 500 µL of a solution of 1 mg mL^−1^ in PBS of the different FITC‐labeled nanoparticles, PEGylated nanoparticles (MSNs‐PA), and coated with PEI (MSNs‐PA@PEI) and our previously reported non‐PEGylated nanoparticles (MSNs), were kept at 37 °C for 1 h without stirring, to be then evaluated under visible and UV light. The suspension stability in 1 mm PBS was further evaluated by DLS to determine the hydrodynamic diameter of the different nanoparticles (MSNs, MSNs‐PA, MSNs‐PA@PEI) after different periods of time (0, 1, 2, 3, 6, and 24 h).

### RNase Stability

The MSNs‐PA@PEI‐siRNA RNase stability was determined by agarose gel retardation assay. Different conditions of siRNA and nanoparticles bound to siRNA were evaluated in order to determine their RNase stability. 1 µg of free siRNA was used as negative control (*ɸ*). Both naked siRNA (1 µg) (Channel 1) and 32 µg of MSNs‐PA@PEI‐siRNA containing 1 µg of siRNA (Channel 2) were incubated with 2 µL of RNase (1 U µL^−1^) in phosphate buffer (pH 7.0) at 4 °C, for 2 h. After time periods, 1 µL of EDTA 100 nm was added to the samples and incubated at 70 °C for 30 min for RNase inactivation. The siRNA was released from the polymers by incubating the sample with 2.5 µL of sodium dodecyl sulfate (SDS) 1% solution at room temperature for 15 min. The extracted siRNA was analyzed on 1% agarose gel electrophoresis carried out at 80 V for 60 min.

### Osteostatin Loading and Release

The MSNs‐PA were loaded before PEI coating with osteostatin (OST) by incubating 5 mg of MSNs‐PA in a solution of OST 10^−4^ m in PBS overnight at 4 °C to ensure osteostatin stability. Then, the nanoparticles were recovered by centrifugation and washed with PBS. The loaded nanoparticles were functionalized with PEI, as previously described. Then, the OST loaded MSNs‐PA@PEI nanoparticles (OST‐MSNs‐PA@PEI) were centrifuged and washed with PBS. A 24‐transwell plate was employed to determine the OST release. From a 14 mg mL^−1^ dispersion of OST‐MSNs‐PA@PEI dispersed in PBS with a pH of 7.4 (10 mm), 0.1 mL were placed on a Transwell permeable support (three replications were performed). The well was filled with 0.6 mL of PBS pH 7.4 (10 mm), and the suspension was stirred at 100 rpm at 37 °C during the experiment. At every time point studied, the solution outside the transwell insert was measured by fluorescence and replaced again on the plate. The amount of cargo released was determined by fluorescence at a wavelength of absorbance/emission of 280/320 nm.^[^ ^68^ ^]^ It was confirmed by a gel electrophoresis assay that osteostatin loading process keeps unaffected the binding ability of OST‐MSNs‐PA@PEI to siGLO.

### Systemic In Vivo Evaluation of OST‐MSNs‐PA@PEI‐siRNA

All procedures were carried out under Project License (Ref PROEX 122/16) approved by the in‐house Animal Care and Ethic Commission for Animal Experiments from the Instituto de Investigación Sanitaria Hospital 12 de Octubre i+12 (Spain) for ERC project (ERC‐2015‐AdG proposal no. 694160). The care and use of animals were performed accordingly with the Spanish law (RD 53/2013) and international standards on animal welfare as defined by European Directive (2010/63/EU) to decrease pain and suffering of the animals. Young mature female C57BL/6J mice (Charles River) that underwent bilateral ovariectomy after 6 weeks of age (OVX) or sham operations (control) of 14 weeks of age were used. They were assigned to 4 groups (*n* = 5 per group), OVX, MSNs‐PA@PEI (MSNs), PTH (PTH), OST‐MSNs‐PA@PEI‐siRNA (OST‐siRNA), and one more group as control (Control) with five healthy mice. They were stabled in the Animal Research Facility at Hospital 12 de Octubre for 2 weeks. All animal experiments in this study were performed according to approved animal protocols. Animals were given free access to water and fed a standard diet (8.8 g per kg calcium and 5.9 g per kg phosphate) in a room maintained at 22 °C on 12 h light/12 h dark cycles.

Mice were anesthetized with isoflurane for every injection. Every 2 days for a period of 2 or 3 weeks, mice were injected with either the different nanoparticle dispersion (MSNs‐PA@PEI; OST‐MSNs‐PA@PEI‐siRNA) (100 µL 0.8 mg mL^−1^), PTH (100 µL), or PBS (100 µL) depending on the group. The injections were performed subcutaneously in the left side of the mice.^[^ ^67^ ^]^ After 2 or 3 weeks of treatment, mice were euthanized by exposing them to 5% isoflurane in oxygen, and both femurs and tibiae were removed.

### Gene Expression Analysis

For the gene expression analysis, one site bone samples were crushed under liquid nitrogen. Total RNA was extracted from these homogenized samples with Trizol (Invitrogen) following the manufacturer's instructions and cDNA synthesis was performed using a high‐capacity RNA‐to‐cDNA kit. Gene expression was analyzed by real‐time qRT‐PCR using a QuantStudio 5 Real‐Time PCR System. Unlabeled mouse‐specific primers for SOST, Runx2, Alp, OPG, OSX, VEGF, and TaqMan MGB probe were used to perform qRT‐PCR assay. The mRNA copy numbers were calculated for each sample by using the cycle threshold (Ct) value. Glyceraldehyde 3‐phosphate dehydrogenase (GAPDH) RNA (a housekeeping gene) was amplified in parallel with tested genes. The relative gene expression was represented by 2−ΔΔC_t_, where ΔΔC_t_ = ΔC_ttargetgene_ − ΔC_tGAPDH_.

### Dual‐Energy X‐Ray Absorptiometry

The bone mineral density of femur was measured in anaesthetized control and ovariectomized mice at the start of the study to confirm decreased bone mass using PIXImus (GE Lunar Corp).

### Micro‐Computed Tomography

The removed femur and tibiae bones were fixed in 10% neutral buffered formalin. Before CT scanning, they were washed with running water for 15 min. The imaging of 3D microcomputed tomography was performed with a CompactCT micro‐CT scanner (SEDECAL). Data were acquired at 27 µm isotropic voxel size with 720 projections by 360° scan, integration time of 2000 ms with three frames, photon energy of 80 KeV, and current of 450 µA. The duration of imaging time was 10 min per scan and followed by projection correction and volume reconstruction of 3D representation. 3D rendered images of the bones were generated through original volumetric reconstructed images by MicroView software (GE Healthcare). The analysis of the results obtained in the microcomputed tomography was quantified from micro‐computed tomography scans using GE MicroView software v2.2.

### Histological and Immunohistochemical Analysis

Fixed femurs were embedded in poly(methylmethacrylate) or in paraffin for histological and immunohistochemical analysis, respectively. For histological studies, hematoxylin/eosin and Goldner trichromic staining were used. All histological determinations were carried out onto sagittal 5–7 mm thick sections/sample in a Zeiss Axio Scope A.1 photomicroscope. For immunohistochemical studies, fixed femurs were subsequently decalcified in Osteosoft, dehydrated, and embedded in paraffin. This analysis was carried out on serial sagittal 4‐mm sections. For sclerostin and Runx2 detection, tissue sections were first incubated in 4% bovine serum albumin in PBS containing 0.1% Triton X‐100 for 30 min at room temperature. The following primary mouse antibodies were used (dilution, fold): rabbit polyclonal anti‐Runx2 antibody (50) and rabbit polyclonal anti‐sclerostin antibody (500). Tissue samples were incubated with the antibodies in a humidified chamber overnight, except for sclerostin which were incubated for 2 h at room temperature. Sections were subsequently incubated with biotinlylated anti‐rabbit IgG and avidinebiotine horse radish peroxidase (HRP) complex or HRPerabbite anti‐rabbit IgG (sclerostin), followed by 3,3′‐diaminobenzidine as chromogen. Sections were counterstained with hematoxylin. Some tissue samples were incubated without the primary antibodies as negative controls. Sclerostin‐positive and total osteocytes were counted in 5 random 400‐fields per sample in a cortical bone segment. Intensity of Runx2 staining was evaluated according to a semiquantitative score, graded from 0 to 3 in 10 consecutive x400‐fields/per sample in the same area. Evaluations were performed by two independent observers in a blinded fashion and the corresponding mean score value was obtained for each mouse.

### Statistical Analysis

Results were expressed as mean ± standard error of mean (SEM). Sample size (*n*) for each statistical analysis was three samples or five animals per group (indicate in each figure). Statistical analysis was performed through nonparametric Kruskal–Wallis test and post hoc Dunn's test with statistical software (SPSS 20, SPSS Inc). All statistical tests were conducted at the two‐sided 0.05 (*p* value) level of significance.

## Conflict of Interest

The authors declare no conflict of interest.

## Supporting information

Supporting Information

## Data Availability

Research data are not shared.
