# Supplementary material for: Osteoporosis Remission and New Bone Formation with Mesoporous Silica Nanoparticles
Source: Adv Sci (Weinh). 2021 Jun 6;8(16):2101107. doi: 10.1002/advs.202101107 (PMC8373152; doi:10.1002/advs.202101107)
Supplement: Supplementary file 1 — Supporting Information [file ADVS-8-2101107-s001.pdf]

© 2021 Wiley-VCH GmbH

ADVANCED SCIENCE
Open Access

Supporting Information

for *Adv. Sci.*, DOI: 10.1002/advs.202101107

Osteoporosis Remission and New Bone Formation with Mesoporous Silica Nanoparticles

Patricia Mora-Raimundo, Daniel Lozano, Manuel Benito, Francisca Mulero, Miguel Manzano, María Vallet-Regí**

Supporting Information

Osteoporosis Remission and New Bone Formation with Mesoporous Silica Nanoparticles

Patricia Mora-Raimundo, Daniel Lozano, Manuel Benito, Francisca Mulero, Miguel Manzano, María Vallet-Regí**

ALN was conjugated via carbodiimide chemistry to the carboxylic acid end of a bifunctional HO_2C-PEG-NH_2 (3500 Da), obtaining the desired targeting molecule (PA) (**Scheme S1**).

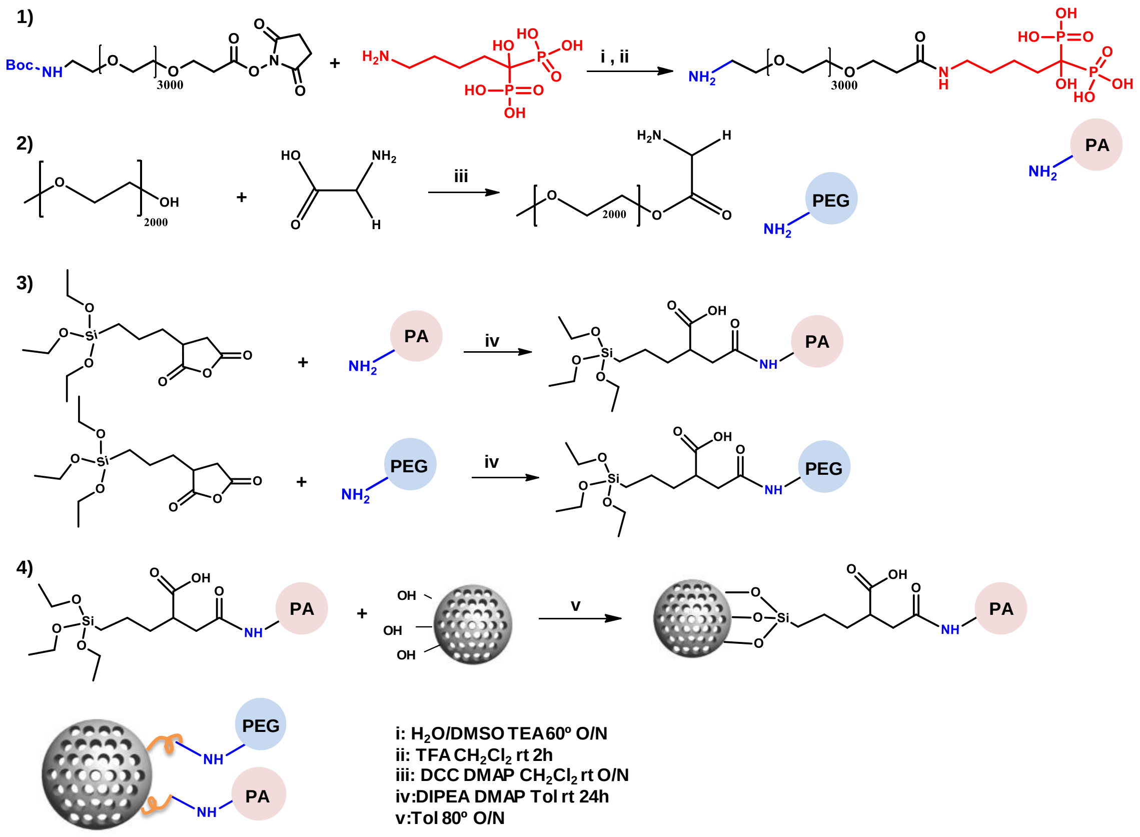

Scheme S1. Scheme of PA and PEG synthesis and nanoparticle functionalization. 1) Reaction between amino group of ALN and activated carboxylic acid of PEG. 2) Reaction between PEG and glycine to obtain amino functionalized PEG. 3) Reaction between both PEG conjugates with 3-triethoxysilylpropylsuccinic anhydride. 4) MSNs surface condensation with the sililated polymers.

The second step regards the obtention of PEG-NH_2. PEG (2000 Da) was conjugated to a residue of glycine to achieve the amino end. The obtention of the final products were confirmed by ^{1}H-NMR (**Figure S1**, **Figure S2**, **Figure S3**).

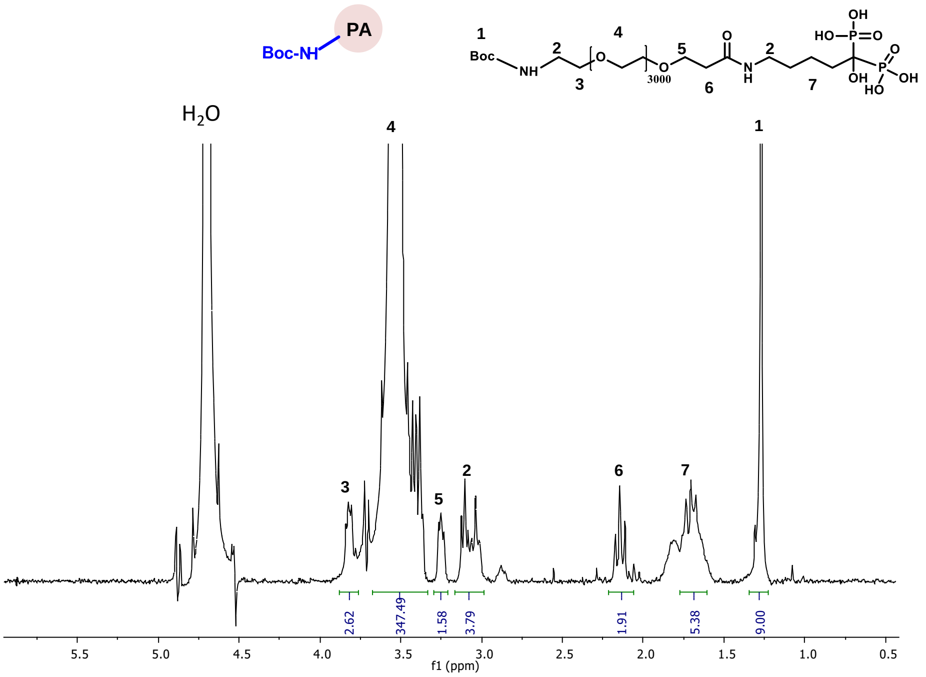

Figure S1. ^{1}H-NMR (D2O) Boc-NH-PA

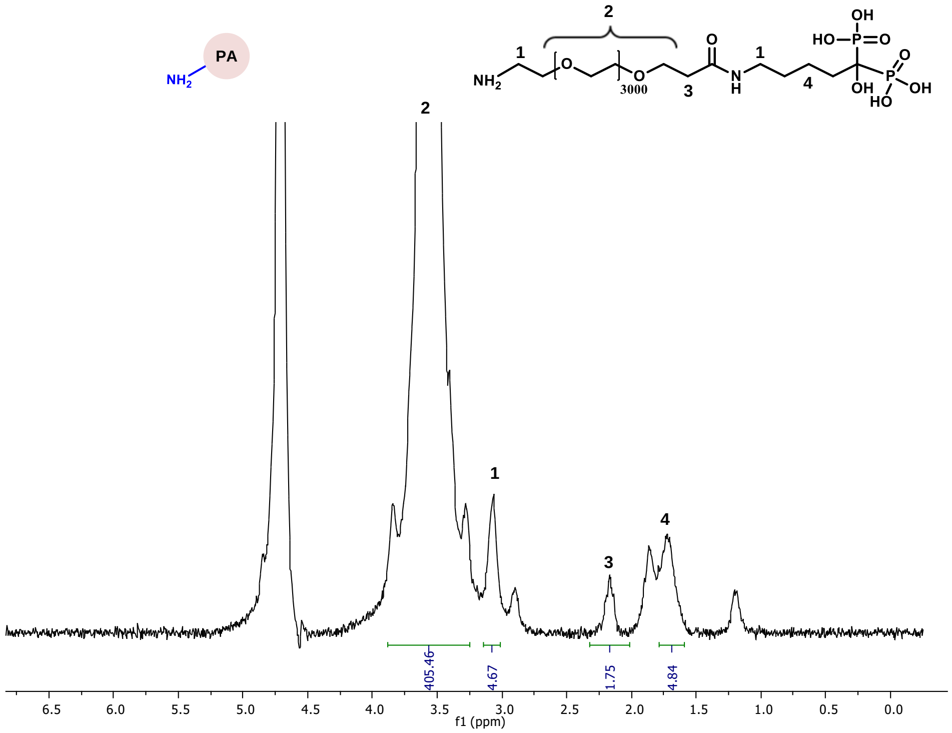

Figure S2. ^{1}H-NMR (D2O) NH_2-PA

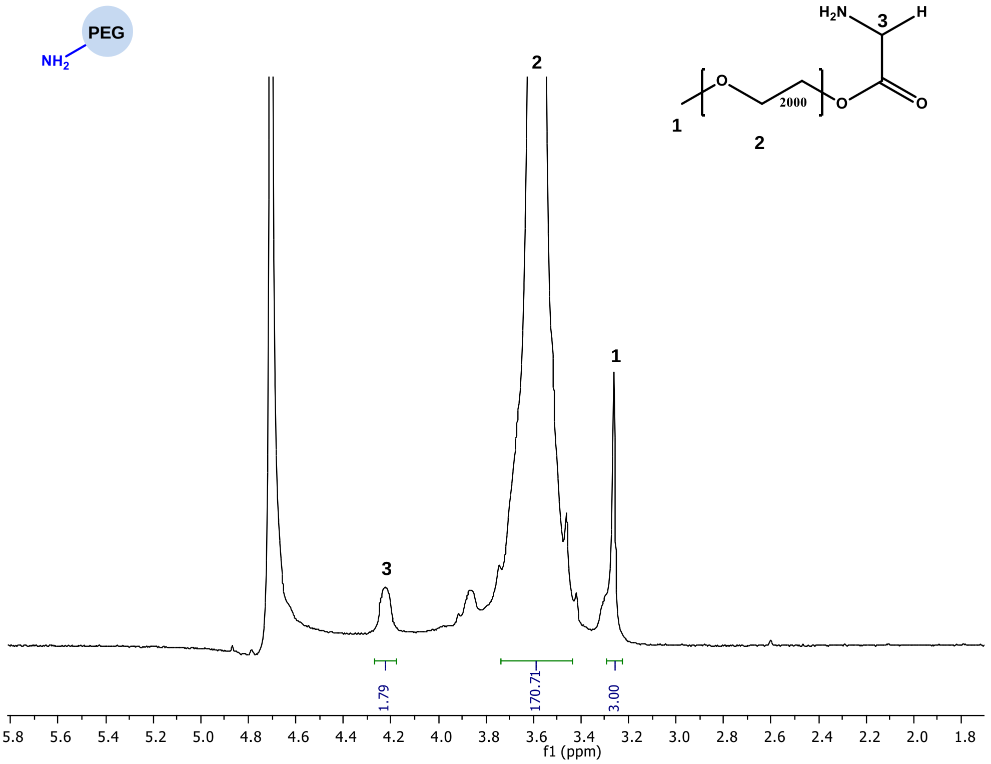

Figure S3. ^{1}H-NMR (D2O) NH_2-PEG

Both conjugates, PEG-NH_2 and PA were then combined (50:50) and condensed to the MSNs surface for obtaining the two different abilities pursued. Both conjugates were linked to 3-triethoxysilylpropylsuccinic anhydride (SATES) by the reaction of the amino group presented in the PEG conjugates and the anhydride group of the silane. Then, the new sililated polymers were added to the nanoparticles for the condensation onto the surface. Finally, the hybrid nanoparticles functionalized with PA and PEG were collected by centrifugation and washed several times with water and ethanol.

The functionalization of the nanoparticles was verified by different techniques (**Figure S4**).

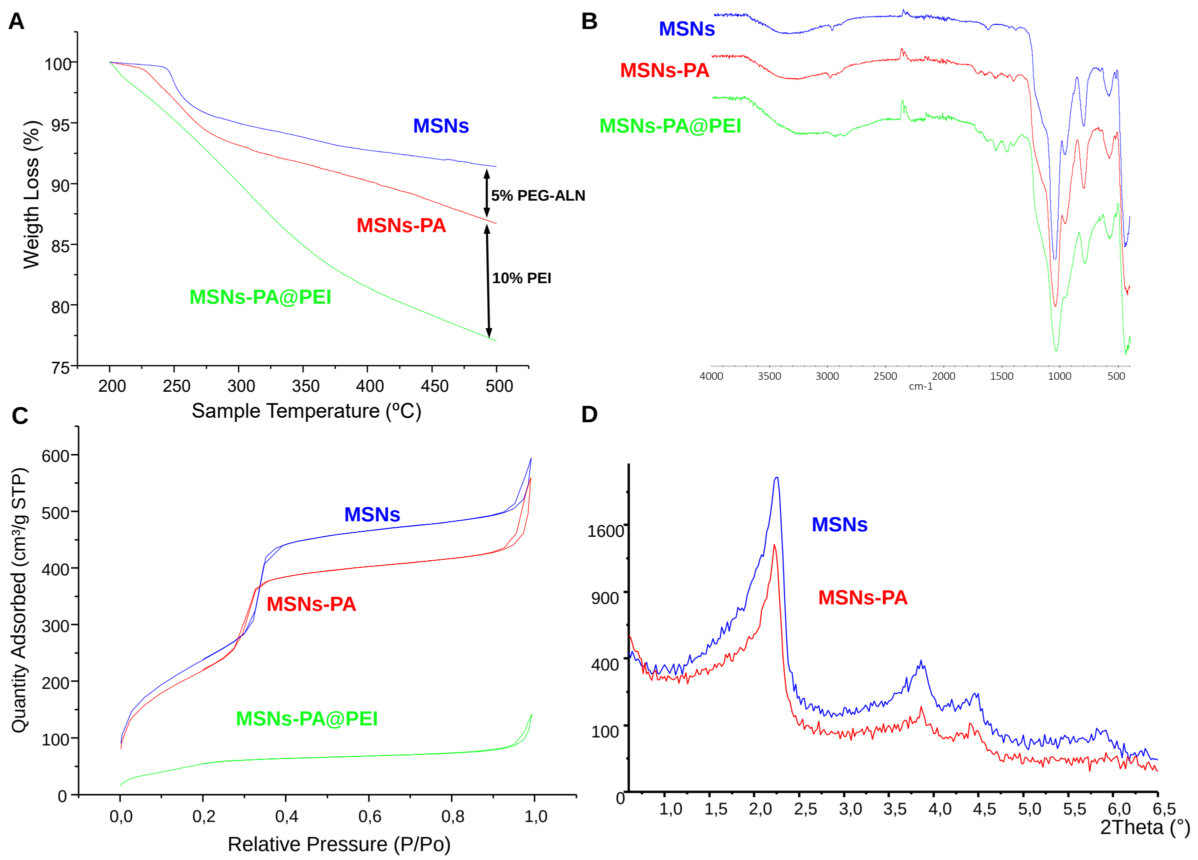

Figure S4. Physicochemical characterization of MSNs. The nanoparticles before (MSNs blue) and after coating with PA (MSNs-PA red) and PEI (MSNs-PA@PEI green) polymer were characterized by (A) thermogravimetric analysis (TGA), (B) Fourier Transform Infrared spectroscopy (FTIR), (C) N_2 adsorption and (D) X-ray diffraction (XRD).

TGA assays confirmed and even quantified the amount of organic matter in the coated nanoparticles. It shows that after the functionalization with PA, a 5% of the weight was organic matter and after PEI grafting the amount increased until 15% of the weight, corresponding 10% to PEI matrix (Figure S4A). In the FTIR spectra it can be observed the presence of silica and the different polymer coatings, PA and PEI. The presence of the typical vibration bands of silica (490-1090 cm^{-1} Si-O-Si) in the naked and coated nanoparticles confirmed the silica frame of the nanocarrier. After coating the MSNs with PA, the presence of the typical vibration bands of PEG (1400 cm^{-1} from the active methylene $-CH_2-CH_2-CO-$ and 2900-2800 cm^{-1} from the C-H from CH_2 groups) could be appreciated. PEI grafting was confirmed through new vibration

bands related with the presence of PEI appeared (3500 cm^{-1} stretching (δ) vibrations of -NH- groups; 2900-2800 cm^{-1} C-H from CH_2 and CH_3 groups; 1600-1400 cm^{-1} bending (ν) vibrations of -NH- groups) (Figure 4SB).

Nitrogen absorption analyses confirmed the porous structure of the material as it can be appreciated in the isotherm plot (Figure S4C). The size of the mesopores was evaluated through the same technique, finding *ca.* 3.4 nm before coating, 3.2 nm and 2.6 after PA and PEI coating respectively. The slight reduction of the surface area, from 895 m^2/g in MSNs to 882 m^2/g in MSNs-PA verified the functionalization of the nanoparticles. However, the reduction of surface area was not really appreciable, then, it could be due to the slight functionalization (5% weight) and/or due to the possible straight conformation (hairy conformation) of the polymer around the surface. On the other hand, after PEI coating, the surface area decreased until 210 m^2/g in MSNs-PA@PEI. This fact also confirmed the successful polymer coating of the nanoparticles, but in this case the reduction was more prominent, meaning that the coating with PEI developed a real polymeric matrix around the particle surface, preventing the access to the pores. The characteristic mesostructure of the MCM-41 type nanoparticles survived the functionalization process as revealed through low-angle XRD where the characteristic diffraction maxima of the 2D hexagonal symmetry were obtained (Figure S4D).

Regarding the results obtained in the electrophoresis gel assay, the siRNA loading capacity of the nanoparticles was found to be *ca.* 3 wt%, and 1.5 wt% for the 1:2 ratio and 1:3 ratio, respectively. Considering that the amount of siRNA recommended by the manufacturer to achieve a knockdown effect was 0.5 μM, the final concentration of siRNA should be *ca.* 6.65 μg/mL. Thus, as the ratio obtained was 32 N/P, it can be approximated that the nanoparticle concentration should be *ca.* 200 μg/mL, which was within the non-toxicity window for MSNs-PA@PEI (1:2 ratio) as appreciated in Figure 3. Then, although the loading capacity of the nanoparticles could be considered low, it was found to be good enough to transport the efficient amount of siRNA needed for an effective knockdown.

The evaluation of the cellular uptake of the MSNs-PA@PEI through flow cytometry was performed in MC3T3-E1 cells (**Figure S5**).

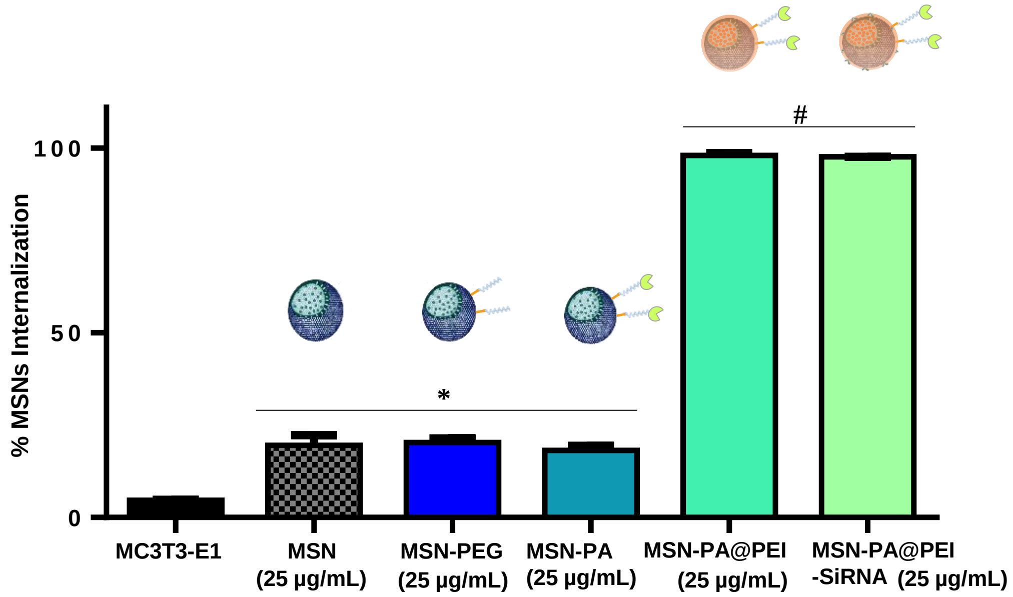

Figure S5. MSNs-PA@PEI-siRNA uptake by MC3T3 cells by flow cytometry. Cellular uptake of different FITC-labeled MSNs, MSNs@PEI, and MSNs@PEI-siRNA was measured by flow cytometry at 2 h of internalization in MC3T3-E1 cells. Data are mean ± SEM of three independent experiments performed in triplicate (n=3). Statistical significance was assessed by Kruskal–Wallis test and post hoc Dunn's test. Asterisks indicate $p < 0.05$ *vs* MC3T3-E1; hashtag indicate $p < 0.01$ *vs* MC3T3-E1, MSN, MSN-PEG and MSN-PA.

Since nanoparticles were functionalized with PEI and PA, it was necessary to test whether the PA functionalization affect the osteostatin release from the MSNs. Osteostatin was loaded into the mesopores of the MSNs-PA by immersing the nanoparticles in an osteostatin solution overnight. Then, the nanoparticles were first washed with PBS to eliminate the extra osteostatin and then coated with PEI. Afterwards, a release experiment was carried out, and the amount of osteostatin released was measured by fluorescence thanks to the presence of tryptophan (Trp) in the pentapeptide of osteostatin (Thr-Arg-Ser-Ala-Trp). In this sense, the indole group of Trp is considered the dominant source of florescence, being excited at ~280 nm and emission at ~350 nm in proteins.[67]

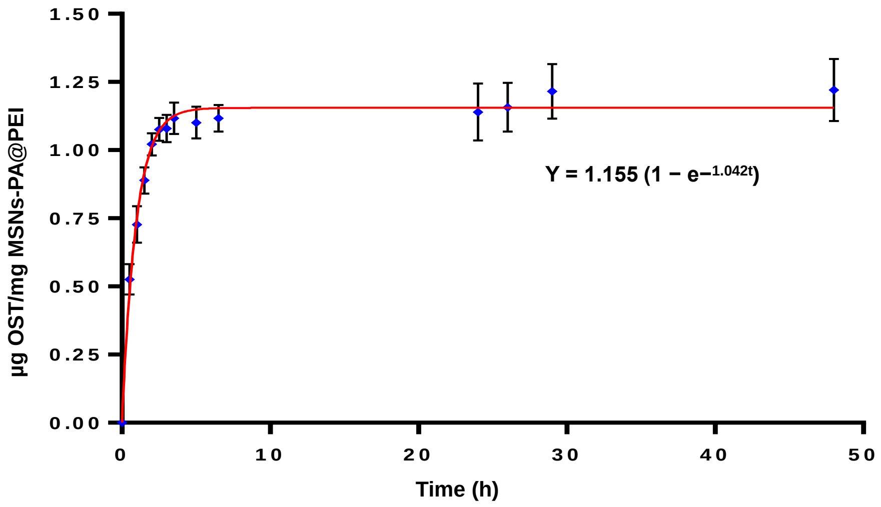

Figure S6. Time-dependent osteostatin (OST) release from MSNs-PA@PEI in PBS at pH 7.4, simulating the physiological fluids. MSNs-PA were loaded with OST and afterwards coated with PEI (OST-MSNs-PA@PEI). Points are the means of three independent measurements per time period (n=3).

The osteostatin release kinetics (**Figure S6**) could be fitted to a first-order kinetic model, with a typical release profile from mesoporous materials (**eq S1**):

$$Y = A(1 - e^{-kt}) \qquad \textbf{eq S1}$$

The equation parameters could be described as Y being the amount of osteostatin released (micrograms of osteostatin per milligram of MSNs-PA@PEI) at time t (h), with A being the maximum amount of osteostatin released (in micrograms of osteostatin per milligram of MSNs-PA@PEI), and with k being the release rate constant.

The release data showed that MSNs loaded with osteostatin and coated with PEI (OST-MSNs-PA@PEI) released osteostatin in a time-dependent manner, reaching values of 1.155 micrograms of osteostatin per milligram of MSNs-PA@PEI. Thus, despite PA functionalization and PEI coating, which are not acting as a physical barrier impeding the osteostatin release, the MSNs were able to release the cargo. Therefore, MSNs-PA@PEI could be used to co-transport and co-deliver osteostatin and siRNA at the same time.

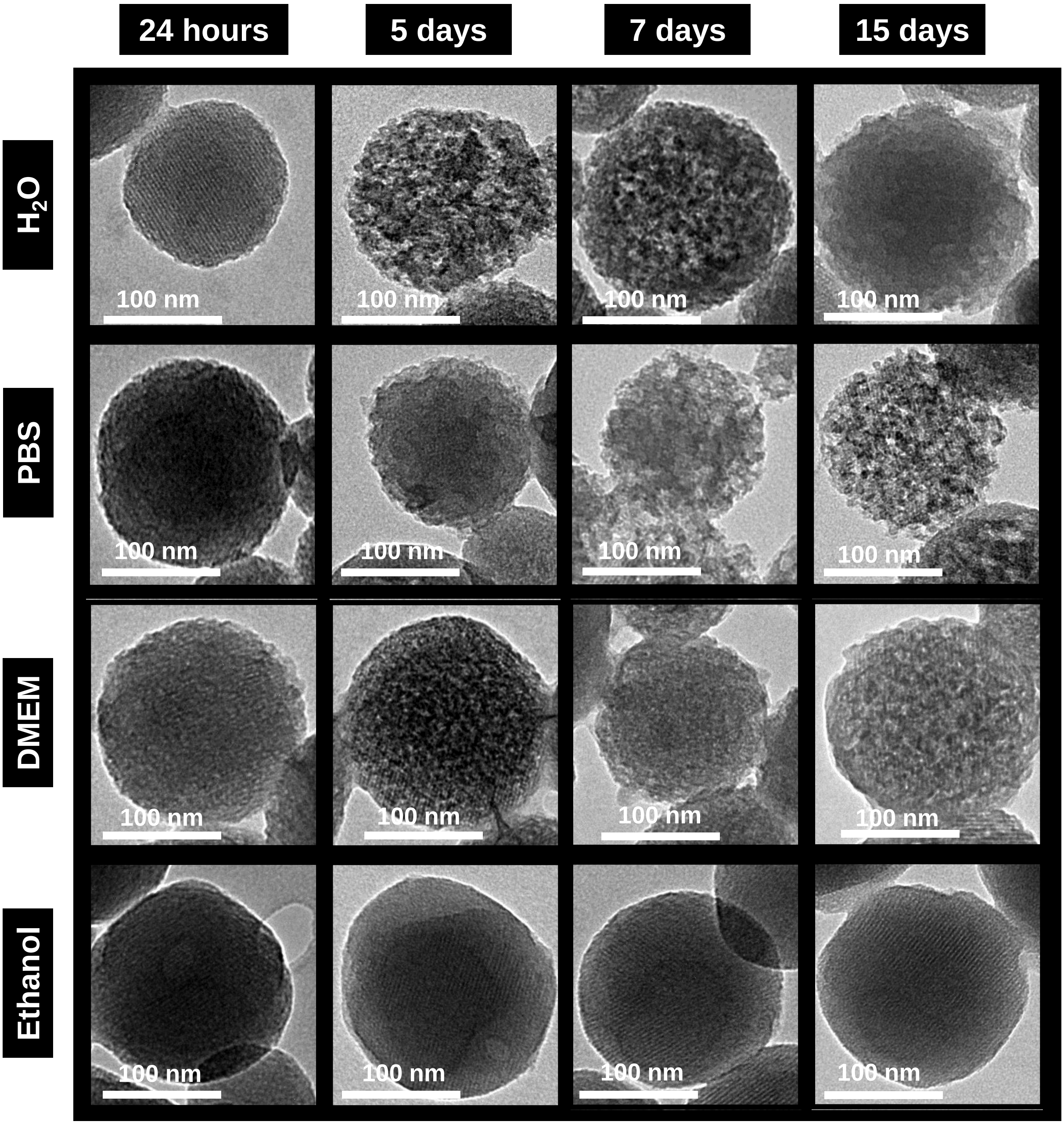

Figure S7. Degradation process of MSNs-PA@PEI in different media (H_2O, PBS, DMEM and ethanol)

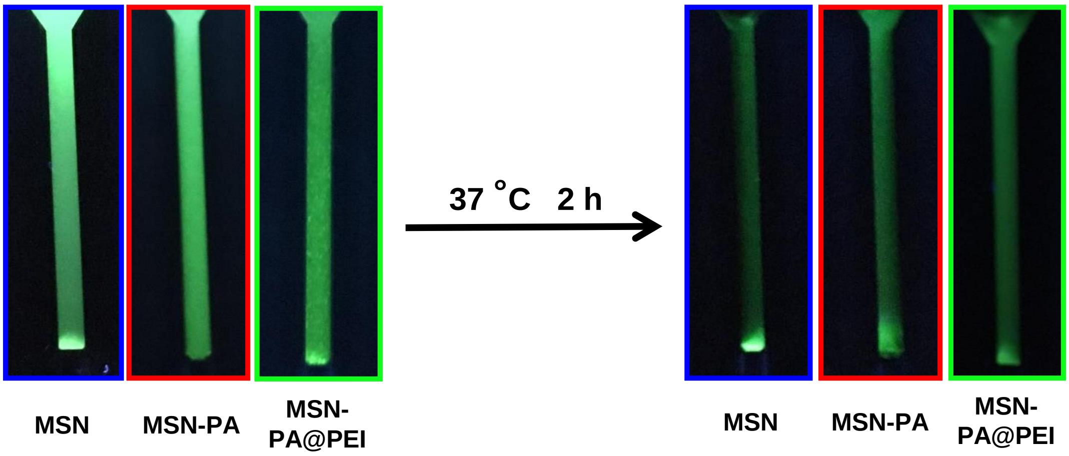

Figure S8. Suspension stability of FITC-labeled MSNs (blue), MSNs-PA (red), MSNs-PA@PEI (green) nanoparticles. Photographs were taken under ultraviolet light.

The particles were dispersed and kept at 37°C for 1 h without stirring and after this time they were evaluated under ultraviolet light. As it can be appreciated in Figure S7, MSNs can be clearly observed at the bottom of the cuvette while MSNs-PA or MSNs-PA@PEI remained dispersed.

To determine the frequency of injections, 100 µL of a cyanine 7 (Cy7) labeled nanoparticles (MSNs-PA@PEI) dispersion (0.8 mg/mL) were injected subcutaneously in the left back (near the leg) of the mice, and evaluated under fluorescence microscopy at different time points (**Figure S9**).

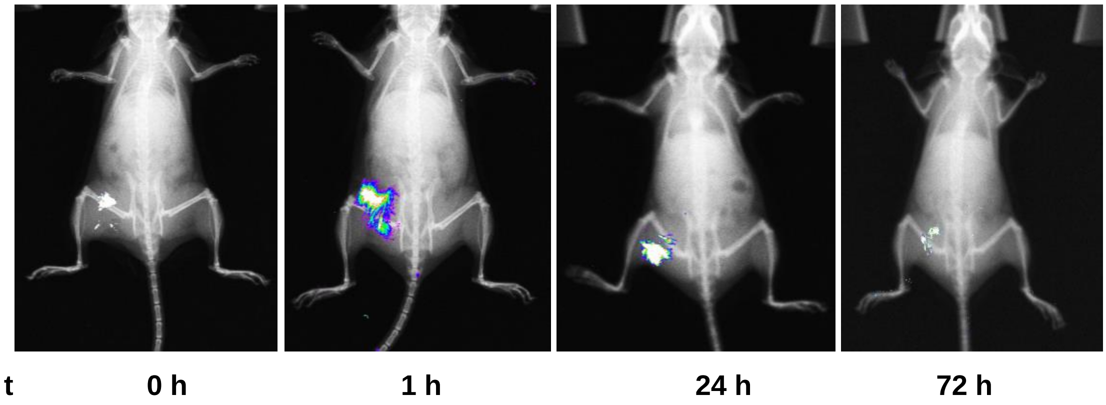

Figure S9. Subcutaneous injection in ovariectomized female mice of cyanine-7 labeled nanoparticles, distribution and accumulation at 0, 1, 24 and 72 hours.

As appreciated in Figure S9, the nanoparticles florescence was decreasing with time but they remained in the organism after even 72 hours, so the injections of the nanoparticles would be performed every two days, to assure presence of nanoparticles at every moment
